# Distribution of metals (Fe, Mn, Zn, Cu) in fish tissues in two lakes of different trophy in Northwestern Poland

**DOI:** 10.1007/s10661-012-2805-8

**Published:** 2012-08-25

**Authors:** Monika Rajkowska, Mikołaj Protasowicki

**Affiliations:** West Pomeranian University of Technology in Szczecin, Szczecin, Poland

**Keywords:** Heavy metals, Microelements, Fish tissues, Bioaccumulation, Correlation, Gender

## Abstract

This study presents concentrations of iron, manganese, zinc, and copper in selected tissues of two fish species: pike (*Esox lucius* L.) and bream (*Abramis brama* L.) living in lakes Ińsko and Wisola, Northwestern Poland. The lakes differ in their trophic status. The effect of gender and environmental conditions on metals accumulation was also investigated. Metal analyses were performed using inductively coupled plasma atomic emission spectroscopy. Considering all studied fish species and tissues, the average metal concentrations (micrograms per gram wet weight) in both lakes occurred in the following ranges: Fe 0.8–240.6, Mn 0.2–8.4, Zn 3.0–185.9, and Cu 0.14–7.76. The lowest levels of the studied metals were always detected in the muscles. The spleen, kidneys, and liver were found to accumulate the highest amounts of Fe. In the case of the other metals, the highest levels were found, as follows: Mn in skin, gills, and gonads, Zn in digestive tract and gills, Cu in liver. Heavy metal content in fish gonads was observed to be sex dependent.

## Introduction

Iron, zinc, manganese, and copper are included in the group of essential trace elements required for maintaining cellular function and are integral components of numerous metal-containing enzymes. However, even essential metals depending on their concentration, may exert beneficial or harmful effects on plant, animal, and human life. Metals enter the aquatic environment from various natural and anthropogenic sources. Contamination of aquatic ecosystems by heavy metals can be confirmed in water, sediment, and organisms (Förstner and Wittman 1983). This is dangerous, because unlike other pollutants (mainly organic), metals are not degraded or eliminated from the ecosystem. Trace metals can be taken up by fish, both through the food chain and from water (Hadson 1988) and ultimately end up in fish, where they accumulate in various organs and tissues. Dietary metal uptake by fish can be as important as waterborne metal uptake and the relative importance of the different uptake routes is variable (Kraal et al. 1995). Metal distribution between different tissues is determined mainly by their content in water and food, and therefore can serve as a pollution indicator of the environment (Farkas et al. 2000). Fish have been largely used in the evaluation of the quality of aquatic systems. These organisms are often at the top of the aquatic food chain and may concentrate large amount of metals from the surrounding waters. Moreover, fish metabolism may be dependent on abiotic conditions, food supply, and the stage of the reproduction cycle (Andres et al. 2000). Microelement content in fish muscle tissues is self-regulated; however, their levels can vary because of certain biological conditions, such as species, sex, age, and feeding (Håkanson 1984; Protasowicki 1986). High intercellular levels of heavy metals can be toxic, resulting in alterations in the intercellular protein machinery, either directly via denaturation of enzymes or indirectly via generation of reactive oxygen species (Pourahmad and O’Brien 2000). The aim of this study was to examine the distribution of Fe, Mn, Zn, and Cu in selected organs of pike and bream from two lakes of different trophy (Lake Ińsko and Lake Wisola) in the northwestern of Poland. The other aim was also examination whether metal levels in fish tissues were related to fish gender, body size, and to environmental levels of metals. Efforts have also been an attempt to identify the most important roads entering the metals to organisms of fish.

## Materials and methods

### Description of the study area

The two investigated lakes are situated in the Ińsko Landscape Park in Northwestern Poland. These lakes are connected by the Ina River, which flows from Lake Ińsko. Lake Ińsko is located in the area of special protection important refuge of birds on European scale Nature 2000. Moreover, there occurs a, rare in Poland, crustacean (*Palasea quadrospinosa*), which is a relic of the Ice Age. We should take care of the metal concentrations in these lakes—especially in fish, because they are an important source of food for birds and other animals that occur in this environment. Based on the authors’ trophic status of exanimate lakes, Lake Ińsko is mezotrophic and Lake Wisola is eutrophic (Kubiak and Knasiak 1996) or Lake Ińsko is α-mesotrophic and Lake Wisola is β-mesotrophic (Filipiak and Raczyński 2000). In the latter case, the classification was based mainly on the oxygen saturation in the hypolimnion, which in α-mesotrophic lake is above 20 % and in β-mesotrophic is below 20 %. The catchment area of Lake Ińsko equals 5.20 km^2^, while Wisola equals 1.73 km^2^ (Filipiak and Raczyński 2000). Lake Ińsko has no point sources of sewage inflow, however, in its south, part there are some holiday centers which constitute a potential risk as sources of pollutions. Lake Wisola is a receiver of mechanically and biologically treated sewage from the Ińsko municipal sewage treatment plant. This lake is distinguished by higher rate of primary production, which is connected with higher biogenic content compared to Lake Ińsko. Lake Ińsko has a high resistance to degradation while Lake Wisola demonstrates low resistance. Fish caught in examined lakes represent a high proportion of fish stock and are sold mainly at the local market.

### Sampling

The fish material was collected eight times, between November 2002 and August 2005, from commercial catches. A total of 80 individuals of pike (*Esox lucius* L.) and 80 individuals of bream (*Abramis brama* L.) were collected from each lake (every season, 10 individuals—five females and five males). The selected species differ in terms of food habits; pike is predator while bream is benthophagus. Pike is a typical predator; it feeds almost exclusively on fish, thus impacting populations of other fish species both quantitatively and qualitatively. A large part of its diet comprises diseased and weakened fish. It can be used to limit the number of undesirable fish species (Paukert et al. 2003). By regulating the population numbers of small planktovorous fish, northern pike can also impact other trophic levels. The diet of breams mainly consists of algae, plankton, insect larvae, pea mussels, crustaceans, molluscs, and other benthic organisms. They are grubbing the bottom sediments to search the many micro-organisms which live there. Both species constitute an important part of commercial fishing in the lakes studied. At the same time, samples of water and sediments were also collected from selected sites on the lakes—eight in Lake Ińsko and seven in Lake Wisola. The results of sediment analysis have been reported by Rajkowska and Protasowicki (2011). All samples were delivered to the laboratory at the day of collection.

### Sample preparation

In the laboratory, the mass and total lengths of the fishes were recorded (Table 1). The fish were dissected in a way that prevented any contamination of the samples. Sex was determined by the inspection of gonads after opening the body cavity. Muscles, gills, liver, kidneys, digestive tract, gonads, spleen, skin, and the content of digestive tract (DTC) were removed from each fish and preserved in the freezer (−20 °C) in clean dry polyethylene bags. To prepare analytical samples, 1 g of selected organs or 2 g of muscles were weighed with an accuracy of ±0.001 g. Every tenth sample was prepared in triplicate. The digestion was performed on hotplate using a mixture (7:3 *v*/*v*) of 65 % nitric acid and 70 % perchloric acid (Merck). After digestion, the samples were dissolved in 5 ml of 15 % HNO_3_ (Protasowicki 1985). In parallel, blank samples and reference materials (DOLT-2 and Fish paste 2) were run in triplicate in each analytical series.

**Table 1:** Mean weight and length of three fish species collected from lakes Ińsko and Wisola

Species	Lake Ińsko				Lake Wisola			
	Weight (g)		Length (cm)^a^		Weight (g)		Length (cm)^a^	
	Mean ± SD	Range	Mean ± SD	Range	Mean ± SD	Range	Mean ± SD	Range
Pike (*E. lucius* L.; *n* = 80)	683 a ± 76	408–1,260	47 a ± 2	40–56	665 a ± 94	263–1,490	47 a ± 2	35–64
Bream (*Abtramis brama* L.; *n* = 80)	708 a ± 272	149–1,560	39 a ± 5	30–51	599 b ± 191	270–1,400	38 a ± 4	30–75

*SD* standard deviation

Mean values marked with different letters indicate a significant difference (*p* < 0.05)

^a^ *Longitudo totalis*

### Determination of Fe, Mn, Zn, and Cu

Concentrations of Fe, Mn, Zn, and Cu were determined using inductively coupled plasma in atomic emission spectrometry (Yobin Yvon JY-24). The quality of the analytical process was controlled by using reference materials: DOLT-2 and Fish paste 2. Recoveries, with respect to the material, were as follows: Fe (88.7 %, –), Mn (89.5, 112.9 %), Zn (85.6, 94.7 %), and for Cu (84.1, 106.3 %). Metal concentrations in organs and tissues were expressed in micrograms per gram wet weight (ww).

### Bioconcentration factor

The bioconcentration factor (BCF) was calculated for each metals, as the relation between the metal concentrations in fish organs (FC) closely associated with water (gills, skin, and digestive tract) and its concentration in water (WC) according to equation: BCF = FC/WC.

### Statistical analysis

Statistics were carried out using Statistica 7.1 software (StatSoft Inc. 2006) and involved analysis of variance. The significant differences between means among tissues and fish species were determined using the Duncan’s multiple range test at the significance level of *p* < 0.05. Furthermore, correlation coefficients were calculated to assess the impact of the environment (water and sediment) and biotic factors (sex, weight, and length) on the concentration of metals in fish.

## Results and discussion

### Metal concentrations in fish tissues

Metal (Fe, Mn, Zn, and Cu) concentrations in fish organs and tissues have been presented in Tables 2 and 3. Metals, in respect of their total amount in examined fish organs and tissues, can be arranged as follows: Zn > Fe > Mn > Cu. Quantitative dependence between the metal content corresponds to the results obtained by Uysal et al. (2008). Considering both studied species and their tissues, the concentration of the investigated elements (micrograms per gram ww) ranged within: Fe 0.8–72.1, Mn 0.2–9.4, Zn 3.0–559, and Cu 0.14–4.61. Metal concentrations in content of digestive tract, besides Mn in skin were higher than in organs (Tables 2 and 3). The results indicate that the concentrations of metals in all fish organs were significantly higher than that in the muscles (*p* < 0.05). This is confirmed in many other studies (Allen-Gil and Martynov 1995; Bervoets et al. 2001; Liang et al. 1999). Liang et al. (1999) demonstrated that higher metal content in viscera was related to their important role in trace metal storage in fish. Many authors (Sobhanardakani et al. 2011; Szarek-Gwiazda and Amirowicz 2006; Bervoets et al. 2001) analyzed metal content mainly in such organs of fish, as liver, kidneys, gills, and muscles. Depending on the metal, they reported the highest accumulation in the liver, kidneys, or gills. Taking into account only these organs, our results are in concordance with authors mentioned above. Szarek-Gwiazda and Amirowicz (2006) pointed on the liver and kidneys as storage organs for Fe, the gills for Mn, and the liver for Cu. In the present study, more organs were analyzed and the highest amounts of the examined metals were observed in the following organs: Fe in spleen, kidneys, and liver; Mn in skin, gills, and gonads, Zn in digestive tract and gills—in case of pike but in gonads and skin in case of bream, and Cu in liver (Tables 2 and 3). According to Morsy and Protasowicki (1990), heavy metals are supposed to be bound by the surface of gills, which affects proper functioning of the organ. We observed high concentrations of Mn and Zn in the gills (Tables 2 and 3). Accumulation of Fe in fish organs in both lakes can be summarized as follows: spleen > kidneys ≈ liver > gills > gonads > digestive tract > skin > muscles. The distribution pattern of Mn in pike and bream organs in both lakes followed the order: skin > gills > gonads > liver > digestive tract > kidneys > spleen > muscles. Zinc in pike was accumulated as follows: digestive tract > gills > skin > kidneys > liver > gonad > spleen > muscles. The same pattern of Zn accumulation was observed by Uysal et al. (2008). Different distribution of Zn was observed in the tissues of bream: skin > gonads > gills > liver > digestive tract > spleen > kidneys > muscles (Table 3). Copper was accumulated in pike as follows: liver > kidneys > digestive tract > skin > gonads > spleen > gills > muscles, in bream: liver > spleen > gonads > digestive tract > kidneys > skin > muscles (Tables 2 and 3). Dural et al. (2006) observed similar distribution pattern of Fe, and Al-Yousuf et al. (2000) reported similar results for Cu, however, unlike in the present study, the same authors found Zn and Mn content in liver to be higher compared to the skin.

**Table 2:** Average concentration of metals in selected organs of pike from lakes Ińsko and Wisola (μg/g ww)

Tissue	Lake Ińsko				Lake Wisola			
	Fe	Mn	Zn	Cu	Fe	Mn	Zn	Cu
Muscles	1.4 **±** 1.8	0.2 **±** 0.1	9.4 **±** 5.2	0.14 **±** 0.06	0.8 **±** 1.2	0.2 **±** 0.2	6.2 **±** 4.4	0.19 **±** 0.16
Gills	24.9 **±** 13.0	2.3 **±** 1.8	170.8 **±** 92.4	0.44 **±** 0.31	20.4 **±** 11.0	2.0 **±** 1.5	163.7 **±** 86.3	0.31 **±** 0.18
Liver	55.9 **±** 36.5	0.7 **±** 0.3	42.5 **±** 25.9	2.64 **±** 1.84	29.7 **±** 15.7	0.7 **±** 0.3	29.1 **±** 14.6	1.87 **±** 1.33
Kidneys	53.1 **±** 21.7	0.3 **±** 0.1	70.5 **±** 36.7	0.51 **±** 0.21	45.4 **±** 23.9	0.3 **±** 0.2	64.4 **±** 29.8	0.60 **±** 0.22
DT	8.6 **±** 5.4	0.8 **±** 0.4	559.0 **±** 203.7	0.52 **±** 0.28	6.5 **±** 3.6	0.7 **±** 0.7	448.3 **±** 121.2	0.51 **±** 0.24
Gonads	9.3 **±** 11.3	1.3 **±** 1.4	34.7 **±** 33.7	0.49 **±** 0.35	5.5 **±** 3.6	1.5 **±** 2.2	43.3 **±** 58.6	0.33 **±** 0.27
Spleen	87.7 **±** 49.3	0.2 **±** 0.1	26.5 **±** 11.0	0.32 **±** 0.13	93.4 **±** 35.7	0.3 **±** 0.1	21.2 **±** 9.2	0.33 **±** 0.17
Skin	3.9 **±** 2.3	5.7 **±** 2.3	115.2 **±** 43.6	0.48 **±** 0.32	3.5 **±** 1.9	6.3 **±** 3.9	102.7 **±** 31.1	0.50 **±** 0.39
DTC	15.8 **±** 12.0	2.2 **±** 1.9	216.8 **±** 124.6	0.92 **±** 0.59	8.0 **±** 5.9	1.5 **±** 1.2	154.8 **±** 92.4	0.52 **±** 0.30

Mean value includes both males and females

*I* Lake Ińsko, *W* Lake Wisola, *DT* digestive tract, *DTC* content of digestive tract

**Table 3:** Average concentration of metals in selected organs bream from lakes Ińsko and Wisola (micrograms per gram ww)

Tissue	Lake Ińsko				Lake Wisola			
	Fe	Mn	Zn	Cu	Fe	Mn	Zn	Cu
Muscles	1.5 **±** 1.8	0.4 **±** 0.4	3.2 **±** 1.9	0.18 **±** 0.15	1.3 **±** 1.1	0.2 **±** 0.2	3.0 **±** 1.6	0.24 **±** 0.15
Gills	24.8 **±** 13.4	8.8 **±** 6.4	12.3 **±** 5.3	0.49 **±** 0.27	47.5 **±** 20.8	9.4 **±** 3.6	19.8 **±** 7.1	0.59 **±** 0.27
Liver	54.9 **±** 32.2	0.8 **±** 0.4	12.3 **±** 5.5	4.07 **±** 2.97	71.3 **±** 71.9	0.8 **±** 0.3	12.8 **±** 7.9	4.61 **±** 3.01
Kidneys	72.1 **±** 44.1	0.6 **±** 0.4	8.2 **±** 4.8	0.53 **±** 0.25	68.3 **±** 46.5	0.6 **±** 0.3	7.5 **±** 5.2	0.49 **±** 0.29
DT	15.3 **±** 9.65	1.4 **±** 1.6	13.1 **±** 7.65	0.80 **±** 0.47	9.4 **±** 5.1	0.7 **±** 0.4	10.6 **±** 8.0	0.57 **±** 0.34
Gonads	7.1 **±** 4.4	2.1 **±** 2.7	26.7 **±** 25.8	0.94 **±** 0.59	8.4 **±** 4.2	2.2 **±** 3.9	29.3 **±** 9.3	0.85 **±** 0.73
Spleen	106.1 **±** 57.8	0.8 **±** 0.8	10.9 **±** 4.7	0.98 **±** 0.56	152.0 **±** 89.5	0.5 **±** 0.3	14.3 **±** 4.7	0.94 **±** 0.41
Skin	4.2 **±** 2.5	8.0 **±** 4.0	36.7 **±** 14.5	0.65 **±** 0.54	3.1 **±** 1.9	8.4 **±** 3.4	46.8 **±** 20.1	0.44 **±** 0.20
DTC	70.8 **±** 58.5	4.0 **±** 4.8	13.1 **±** 7.4	1.24 **±** 1.19	53.1 **±** 37.1	2.9 **±** **±** 5.0	11.5 **±** 7.6	0.86 **±** 0.59

Mean value includes both males and females

*I* Lake Ińsko, *W* Lake Wisola, *DT* digestive tract, *DTC* content of digestive tract

Ptashynski et al. (2002) indicated that metal absorption in fish is carried out via two uptake routes: digestive tract (dietary exposure) and gill surface (waterborne exposure). Food chain was recognized as the most important road of entering Cu to organisms of bream, as evidenced by positive correlations between Cu concentration in the kidneys or spleen, and in the digestive tract (*r* = 0.5–0.78). Such observations were also observed between Fe concentration in the skin and Fe in the liver (*r* = 0.50–0.53) in pike from both lakes. The concentration of Fe in the digestive tracts was positively correlated with its participation in the kidneys (*r* = 0.54–0.59) of pike. Positive correlation was found between Fe in the gills and gonads of bream from Lake Ińsko (*r* = 0.50). These relationships show that Fe is taken up by the digestive system, skin, and gills of fish.

Statistical analysis (Duncan’s test) confirmed that in Lake Ińsko, there were smaller, or none as in case of Cu, interspecific differences in metal accumulation in fish organs than in Lake Wisola (Table 4). The observed differences were mainly associated with the accumulation of metals in organs such as gills, liver, kidneys, and also in the contents of digestive tract. In both lakes, there were little or no significant interspecies differences in average metal concentration in the gonads, spleen, digestive tract, and muscles. Zinc concentration in pike organs were significantly higher than in bream. The results obtained by Campbell (1994) indicate that predators accumulate more zinc than benthivores. The opposite situation was in the case of Fe, Mn, and Cu content in some tissues of fish (Tables 2 and 3).

**Table 4:** The comparison of the species with respect to the metal accumulation levels of the tissues

Material	Significant differences between fish species (*p* < 0.05)							
	Lake Ińsko				Lake Wisola			
	Fe	Mn	Zn	Cu	Fe	Mn	Zn	Cu
Muscles	–	–	–	–	–	–	–	–
Gills	–	P < B	P > B	–	P < B	P < B	P > B	P < B
Gonads	–	–	–	–	–	–	–	P < B
Kidneys	P < B	–	P > B	–	P < B	P < B	P > B	
Liver	–		P > B	–	P < B	P < B	P > B	P < B
Spleen	–	–	–	–	–	P < B	–	P < B
DT	–	–	P < B	–	–	P < B	P < B	–
Skin	–	P < B	P > B	–		P < B	P > B	–
DTC	P < B	P < B	P > B	–	P < B	P < B	P > B	P < B

*DT* digestive tract, *DTC* content of digestive tract, *–* no significant interspecific differences, *P* pike (*E. lucius* L.), *B* bream (*A. brama* L.)

### The effect of body size and gender on metal content in fish organs

Significant differences (*p* < 0.05) between both lakes were observed in body weight of sampled breams (Table 1). The fish from Lake Ińsko were bigger than those from Lake Wisola. In the gonads of pike from Lake Ińsko, Fe content was positively correlated (*p* < 0.05) with body length (*r* = 0.76), while Zn content correlated with both length and weight (body size; *r* = 0.69, *r* = 0.75). In the pike from Lake Wisola, body size was positively correlated with Fe content in the gills (*r* = 0.59, *r* = 0.73), whereas in case of bream such dependence was observed for Fe and Cu in the kidneys and liver (*r* = 0.62, *r* = 0.81). Dependence of Cu concentration in liver from body size was previously observed by Farkas et al. (2003). Liang et al. (1999) observed that Cu concentration in viscera deceased with increasing body length of fish. The same authors showed different Zn accumulation pattern in viscera, which increased with increasing body length and decreasing Zn content in flesh. Farkas et al. (2003) observed that the concentration of Zn significantly decreased with increasing length. Devieller et al. (2005), who analyzed the muscle and liver of fish, observed only weak or none effect of fish length on the metal content in tissues.

**Fig. 1:**
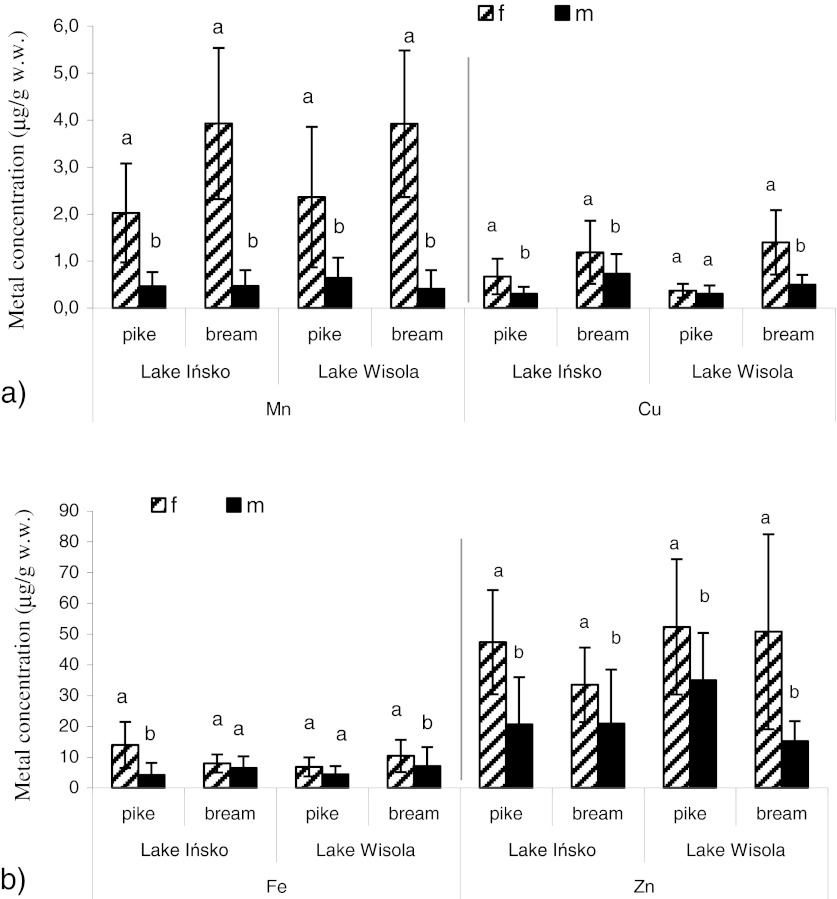
Significant differences in Fe, Zn (**a**), Mn and Cu (**b**) accumulation in fish gonads between female (*f*) and male (*m*) in Lake Ińsko and Lake Wisola. *Means sharing the same letters* were not significantly different between *f* and *m* of fish (*p* < 0.05, Duncan’s test comparison of means)

Gender differences in metal tissue levels may be influenced by a combination of factors, such as dietary preferences, physiological metabolism in relation to stage in the reproductive cycle or foraging behavior (Alquezar et al. 2006). In this study, gender did not exert a significant effect on metal concentrations in most organs of fish, however, the concentration of Mn and Cu in fish gonads showed considerable differences between males and females—mainly in Lake Ińsko (Fig. 1a). In both fish species, female gonads accumulated higher amounts of the examined metals compared to male reproductive organs. These differences were less significant in the case of Fe and Zn (Fig. 1b). In the studies of Protasowicki (1986), the gonads of females contained about five times more Zn than those of males. The author of the study explains that the higher amounts of indispensable metals in female gonads confirm the importance of these elements in fish embryonic development. No gender differences in metal bioaccumulation in red mullet and hake were previously reported by Gaspic et al. (2002). Al-Yousuf et al. (2000) however found higher average metal concentrations in the liver, skin, and muscle of female fish compared to male fish.

### Environmental impact on metal accumulation in fish

Table 5 presents the concentration of the metals in water and bottom sediments of lakes studied. Metal concentrations in water were used to calculate BCFs, which indicated that among examined metals Zn was the most readily absorbed by fish (Table 6). The presented research has shown also that water was a good source of Fe, which penetrated into fish organisms mainly through the gills. Manganese was absorbed through the gills and skin of bream and the skin of pike. Depending on the fish species, Zn and Cu showed the highest affinity to: digestive tract, skin, and gills (Table 6). Uysal et al. (2008) observed the highest BCFs for Mn in the gills and Zn in the intestine. For both fish species, the BCFs for Fe, Mn, and Zn were higher in Lake Ińsko, while the BCFs for Cu were higher in Lake Wisola. The BCFs obtained in this study were comparable to those reported by Salánki et al. (1982). Positive correlation coefficients (*p* > 0.05) were obtained between Fe concentrations in water and in the muscles of pike from Lake Ińsko (*r* = 0.74). Manganese in the muscles and digestive tract of bream from Lake Ińsko and in the digestive tract of pike from Lake Wisola was positively correlated with its concentrations in water (*r* = 0.51–0.86, *r* = 0.53–0.61, respectively). Positive correlations of Mn in fish organs with its concentration in water were previously observed by Moiseenko and Kudryavtseva (2001). Bervoets et al. (2001) found correlations between zinc in gills and its concentration in water and between copper concentration in muscles and gills and its content in sediment and water. In the present study, positive correlations occurred between Cu concentration in the digestive tract of bream from Lake Ińsko and its concentration in water (*r* = 0.78–0.94). Furthermore, in Lake Ińsko, concentration of Cu in the digestive tract of examined fish was positively correlated with its total concentration in sediments and its content in the skin with bioavailable fractions of Cu in sediments (Table 7). It is in good agreement with results indicated by Rajkowska and Protasowicki (2011) who showed that in both examined lakes, Cu occurred in the highest proportion in the mobile fractions even up to 37 % was in the exchangeable fraction. Bochenek et al. (2008) observed relationships between Cu and Zn concentration in roach tissues and the sediments. They also find correlation between copper concentrations in kidneys and in the carbonate fraction of sediments. According to Fan et al. (2002), extraction and disturbance processes altering redox potential of sediments and chemical forms of heavy metals can accelerate the emission flux of heavy metals from sediments to water and organism. Because of breams diet mainly consists of invertebrates—chironomid larvae and other benthic organisms—and the metal load of the water column of the sampling sites proved to be relatively low (Table 5), the metal levels detected in organs of bream seem to reflect the pollution level of the sediment and its biota, rather than the prevailing pollution state of the water. In such conditions, according to previous studies (Farkas et al. 2002), the metal uptake from food is predominant.

**Table 5:** Metal concentrations in water and sediments from lakes Ińsko and Wisola in period 2002–2005

Material	Lake	Mean ± SD			
		Fe	Mn	Zn	Cu
Water (μg/l)	Ińsko	21.8 ± 16.3	8.5 ± 6.1	10.0 ± 6.1	0.66 ± 0.55
	Wisola	29.7 ± 21.3	25.1 ± 21.0	17.1 ± 9.3	0.51 ± 0.39
Total sediment^a^ (μg/g dry weight)	Ińsko	21,840 ± 4,675	256 ± 35	138.3 ± 21.2	17.5 ± 3.4
	Wisola	13,774 ± 3,429	189 ± 63	149.6 ± 57.0	19.5 ± 6.7

^a^Data published by Rajkowska and Protasowicki (2011)

**Table 6:** BCF as the ratio of mean metal content in selected fish tissues to its mean concentration in water

Metal	Tissue*	Pike (*E. lucius* L.)		Bream (*A. brama* L.)	
		Lake Ińsko	Lake Wisola	Lake Ińsko	Lake Wisola
Fe	Digestive tract	394	219	702	316
	Skin	179	118	193	104
	Gills	1,142	687	1,138	1,599
	Muscles	64	27	69	44
Mn	Digestive tract	94	28	165	28
	Skin	670	251	941	335
	Gills	270	80	1,035	374
	Muscles	23	8	47	8
Zn	Digestive tract	55,900	26,216	1,310	620
	Skin	11,520	6,006	3,670	2,737
	Gills	17,080	9,573	1,230	1,158
	Muscles	940	363	320	175
Cu	Digestive tract	788	1,000	1,212	1,118
	Skin	727	980	985	863
	Gills	667	608	742	1,157
	Muscles	212	372	273	470

*Only muscles and organs directly associated with waterborne metals

**Table 7:** Correlation coefficient between metal levels in fish tissues and their total and different geochemical fractions content in bottom sediments (*p* < 0.05)

Tissue^*^	Species	Correlation coefficient (*r*)	
		Lake Ińsko	Lake Wisola
Gills	Bream	Fe_IV_ 0.96; Fe_V_ 0.87; Mn_T_0.92; Mn_IV_ 0.91	Zn_V_ 0.94
Digestive tract	Pike	Mn_III_ 0.82 Mn_I_ 0.90; Mn_III_ 0.65; Mn_V_ 0.88; Zn_T_ 0.76; Zn_I_ 0.73; Cu_T_. 0.63	Fe_III_ 0.91 Zn_II_ 0.68
	Bream	Zn_T_ 0.94; Zn_I_ 0.96; Cu_T_0.93	Fe_III_ 0.60; Mn_I_ 0.83
Skin	Pike	Mn_II_ 0.75; Mn_III_ 0.68	Mn_IV_ 0.64; Mn_V_ 0.68
	Bream	Fe_T_0.88; Cu_I_ 0.98; Cu_II_ 0.93	Fe_III_ 0.75; Zn_II_ 0.97; Zn_III_ 0.89
Muscles	Pike	Fe_III_ 0.64	–
	Bream	Fe_IV_ 0.87; Fe_V_ 0.85	Fe_II_ 0.83; Mn_IV_ 0.78; Zn_II_ 0.99; Zn_III_ 0.94; Cu_V_ 0.79

*The data relate only to the muscles and organs, which are in direct contact with sediment; *Me*_*T*_ total content of the element in the bottom sediments; *Me*_*I*_ *–Me*_*V*_ metal content in different geochemical fractions: *I* exchangeable, *II* carbonate, *III* easily reducible, *IV* medium reducible, *V* sulphidic organic (Rajkowska and Protasowicki 2011)

## Conclusions

There were no significant differences on metal concentrations in organs of fish between the same species from two lakes. It could be concluded that small differences in trophy level does not influence metal bioavailability to fish. The results however indicate that the concentration of trace metals in fish vary significantly among two investigated species. In most organs and tissues of bream, the concentrations of Fe, Mn, Cu were higher than in pike and vice versa in case of Zn. Significant effect of sex on the metal content in the gonads of fish was observed. As demonstrated by the correlation coefficients, the concentration of metals in bream depended on their concentration in the bottom sediments of lakes surveyed. Taking into account feeding behavior of examined species, we could conclude that sediments and its biota are the main sources of metals in the bream diet.
